# Highly-Loaded Thermoplastic Polyurethane/Lead Zirconate Titanate Composite Foams with Low Permittivity Fabricated using Expandable Microspheres

**DOI:** 10.3390/polym11020280

**Published:** 2019-02-07

**Authors:** Gayaneh Petrossian, Cameron J. Hohimer, Amir Ameli

**Affiliations:** Advanced Composites Laboratory, School of Mechanical and Materials Engineering, Washington State University, 2710 Crimson Way, Richland, WA 99354, USA; Gayaneh.Petrossian@wsu.edu (G.P.); Cameron.Hohimer@wsu.edu (C.J.H.)

**Keywords:** piezoelectric, functional foam, piezocomposite, PZT, expandable microspheres, permittivity

## Abstract

The sensitivity enhancement of piezocomposites can realize new applications. Introducing a cellular structure into these materials decreases the permittivity and thus increases their sensitivity. However, foaming of piezocomposites is challenging because of the high piezoceramic loading required. In this work, heat-expandable microspheres were used to fabricate thermoplastic polyurethane (TPU)/lead zirconate titanate (PZT) composite foams with a wide range of PZT content (0 vol % to 40 vol %) and expansion ratio (1–4). The microstructure, thermal behavior, and dielectric properties of the foams were investigated. Composite foams exhibited a fine dispersion of PZT particles in the solid phase and a uniform cellular structure with cell sizes of 50–100 μm; cell size decreased with an increase in the PZT content. The total crystallinity of the composites was also decreased as the foaming degree increased. The results showed that the relative permittivity (*ε_r_*) can be effectively decreased by an increase in the expansion ratio. A maximum of 7.7 times decrease in *ε_r_* was obtained. An extended Yamada model to a three-phase system was also established and compared against the experimental results with a relatively good agreement. This work demonstrates a method to foam highly loaded piezocomposites with a potential to enhance the voltage sensitivity.

## 1. Introduction

Over decades, great attention has been paid to functional polymer composites, such as conductive [1,2], piezoresistive [3], and piezoelectric [4,5] composites, as they combine the advantages of the constituent materials. Piezoelectric materials are used as sensors in medical, automotive, and aerospace industries. These materials are mainly divided into four categories: piezoceramics [6,7], piezopolymers [8,9,10], ferroelectrets [11,12], and piezoceramic-polymer composites [4,5]. The conventional piezoelectric sensors are mainly made of piezoceramics, notably lead zirconate titanate (PZT) and barium titanate (BTO). Although these materials can have large piezoelectric charge constants (d), they are very brittle and difficult to shape into mechanically compliant structures [13]. On the other hand, polymers can be ductile, easy to shape, and low in material and processing cost. Therefore, piezopolymers, such as polyvinylidene fluoride (PVDF) and its copolymers and ferroelectrets (also known as piezoelectrets), were developed mainly to address the limitations of piezoceramics. Piezoelectrets are electrically charged polymer foams, mostly made of polypropylene. However, their weak piezoelectric response and low thermal stabilities, together with limited flexibility, limits their applications [14].

Recently, piezoceramic–polymer composites, also known as piezocomposites, have been paid significant attention as the intermediate materials between the two extremes, in an attempt to obtain a more desirable combination of structural and functional properties [15,16,17]. Even though the piezoelectric coefficient of the resultant composite will be lower than that of the ceramic constituent, combining the ceramic’s high piezoelectric coefficient and the desirable mechanical properties and processing capability of polymers [18] provides a means of designing composites with desirable manufacturing and performance characteristics for a range of applications. Among polymers, thermoplastic polyurethane (TPU) is a highly flexible material and is desirable as a matrix for applications where conformability is a priority. It is a block copolymer, composed of soft and hard segments, providing the possibility to tune its elasticity [19]. Because of the high flexibility, low modulus of elasticity, and recoverability, TPU behaves similarly to elastomers, but at the same time, remains thermoplastic, which is melt-processable and recyclable, lending itself to more scalable manufacturing processes such as injection molding. The low stiffness of TPU as a matrix facilitates the large mechanical deformation of the composite. In addition, it counteracts the brittleness of the ceramic filler, such that the resultant composite at high loads of piezo particles may still be deformable repeatedly under large strains. However, in piezocomposites with random alignment of fillers, because of the high ceramic filler loadings required, the resultant composite is still rigid and heavy and exhibits relatively low piezoelectric voltage constant (*g* = *d*/*ε* [13]), which is the primary performance indicator of a piezoelectric material for sensing applications. The low *g* is associated with the high permittivity (*ε*) of PZT and the low *d* of the polymer matrix. 

Foaming of functional polymer composites has been studied to enhance their performance, develop lightweight products, and explore new applications [2,20,21]. Some works have demonstrated the ability of foaming to enhance the electrical properties [1], dielectric properties [22], and electromagnetic interference (EMI) shielding effectiveness (SE) [2] of polymer composites. Cellular structure can also potentially enhance the touch sensitivity of piezocomposites. In the past, several methods have attempted to enhance the sensing efficiency of piezocomposites [23,24,25,26]. The introduction of cellular structure to piezocomposites can result in a decreased permittivity (*ε*) [27,28,29,30]. As reported in the work of [31], the introduction of cellular structure will not affect the overall value of *d*, and thus the decreased permittivity should enhance the sensing capability (*g*). Another appealing characteristic of foamed materials is their enhanced mechanical flexibility. Polymer foams are composed of a polymeric matrix and gas inclusions to form a cellular structure with a lower density, which inherently enhances the mechanical conformability of the resultant cellular structure. This feature can be of great importance for flexible applications. Therefore, the combination of foaming and the TPU/PZT composite will potentially offer higher *g* values and greater flexibility and conformability compared with the unfoamed piezocomposite counterpart.

Very limited work, however, has been reported on the foaming of piezocomposites. Recently, De Boom et al. [27] and Khanbareh et al. [28,29] reported polyurethane/PZT composite foams prepared using magnetic stirring and the whipped cream maker method. McCall et al. [30] worked on polydimethylsiloxane (PDMS)/BTO/multiwalled carbon nanotube (MWCNT) composites and foamed them via the sugar-templating method. In all the cases, either thermosetting polymers are used or the foaming methods are not scalable to current industrial fabrication processes. To further broaden the applications, scalable methods and new material compositions need to be developed for highly flexible and sensitive piezocomposites. Here, we have investigated a facile and scalable method of preparing cellular structures in thermoplastic-based piezocomposites with high loadings (up to 40 vol %) that can eventually be scaled to industrial fabrication processes, such as injection molding.

Physical foaming with the aid of a gas such as nitrogen or carbon dioxide is the environmentally benign method of foaming at the industrial scale. However, polymers filled with a high content of dense additives such as PZT exhibit significantly decreased physical foaming ability. This is attributed to difficulty in gas diffusion, decreased fraction of the matrix available, the reduced number of nucleated cells, and the difficulty in cell growth. In the physical foaming using CO_2_, Matuana et al. [32] reported that an increase in wood flour loading from 0 vol % to 40 wt % in polylactide (PLA)/wood flour composite foams decreased the expansion ratio from 10 (for neat PLA) to 2 (for PLA/40 wt % wood flour). They also reported that increasing the filler loading beyond 10 wt % resulted in poorly foamed composites. One possible alternative for gas foaming is to use expandable microspheres, which are thermoplastic beads encapsulating a liquid hydrocarbon. Upon heating, the liquid gasifies and expands the microspheres. Using microspheres, Chan et al. [33] could foam polyethylene composites loaded with up to 50 vol % boron nitride to achieve lightweight and thermally conductive materials. 

In this work, we report highly loaded TPU/PZT foams with high foaming degrees to achieve lightweight and flexible composites with significantly decreased dielectric permittivity. Piezocomposite foams with 0 vol % to 40 vol % PZT loadings and expansion ratios of 1–4 were fabricated using expandable microspheres as the foaming agent. The physical, morphological, thermal, and dielectric properties of the resultant foams are characterized and the effects of the expansion ratio are explained. It is shown that the permittivity of the TPU/PZT composite is effectively decreased over a range of PZT contents and expansion ratios. Furthermore, the experimental data are compared with those of the theoretical extended Yamada model, and some predictions are established for piezocomposite foams.

## 2. Materials and Methods

### 2.1. Materials

The commercially available TPU (Elastollan^®^ 1185A, BASF Ltd., Wyandotte, MI, USA), having a density of 1.12 g.cm^−3^, a glass transition temperature of −38 °C, and *ε_r_* of 6.4, was used as the base resin. The PZT powder, with an average particle size of 1 μm and *ε_r_* of 1850, was supplied by Hammond Lead Products, Hammond, IN, USA. Liquid nitrogen was purchased from Oxarc Company (Spokane, WA, USA) and used in differential scanning calorimetry (DSC) tests. The expandable microspheres were grade Expancel 980 DU 120, supplied by Akzonobel, Duluth, GA, USA. This grade has an initial particle size of 25–40 μm and a density of 1.00 g.cm^−3^. The activation temperature starts from 158 to 173 °C (*T*_start_) and can be increased to a maximum of 215–235 °C (*T*_max_). Tetrahydrofuran (THF) was purchased from VWR, Radnor, PA, USA, and used as the solvent. All materials were used as received. All the properties reported in this section were provided by the manufacturers. 

### 2.2. Preparation of TPU/PZT Composites

**Figure 1:**
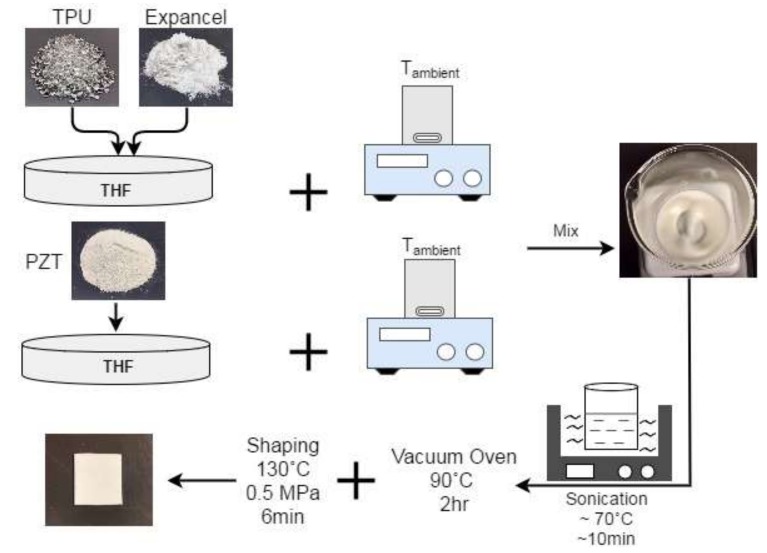
Schematic illustration of the solution casting process. TPU—thermoplastic polyurethane; PZT—lead zirconate titanate; THF—tetrahydrofuran.

The composites were made using a solution casting method (Figure 1). First, TPU pellets were oven-dried at 90 °C for 2 h to remove any excess moisture. TPU pellets and the expandable microsphere powder were then mixed together and dissolved in THF using a magnetic stirrer. The microspheres’ loading was fixed at 5 wt % of the composite. PZT powder was dispersed separately in THF with a magnetic stirrer. The PZT slurry was then added to the TPU/microsphere mixture. The PZT content in the composites were fixed at 10–20–30–40 vol % of the composite. The mixtures were then magnetic stirred to obtain a viscos slurry and put into an ultrasonic bath, with a water temperature of 70–80 °C. The sonication at elevated temperature prevented the fillers from precipitating and accelerated the evaporation process. The samples were then dried in a vacuum oven for 2 h at 90 °C for complete removal of THF. The samples were then hot-pressed (Carver Inc., Wabash, IN, USA) at 130 °C and 0.5 MPa for 6 min. In order to prevent the samples from foaming at this stage, 130 °C was selected for shaping the samples, which is lower than the *T*_start_ of expandable microspheres. This generated a solid layer of the composite with unexpanded microspheres.

### 2.3. Fabrication of TPU/PZT Foams

Foaming was conducted using a hot press as the heat source. The composite samples were placed between the two platens of the press at 160 °C and 0.5 MPa for 1 min. The pressure was then released to allow the composite expansion, while the samples remained at the elevated temperature between 1 and 4 min to assure that the viscosity of the samples was low enough to allow microsphere expansion. The samples were then removed from the hot press and left outside for 1–5 min for further expansion. After the desired foaming degree was achieved, the samples were quenched in cold water to prevent further expansion. Different foaming times were used to obtain a range of the expansion ratio. It is noted that for foamed samples, the reported PZT volume contents are all based on the initial volumes, not on the final volumes after foaming, unless otherwise stated. 

### 2.4. Characterization

The microstructure and cellular morphology of the TPU/PZT foams were examined using a JEOL JSM-6060 scanning electron microscope (SEM) (JEOL USA Inc., Peabody, MA, USA). The samples were cryo-fractured using liquid nitrogen before sputter coating. The densities of the solid (*ρ_u_*) and foamed (*ρ_f_*) composites were measured using the water-displacement method (ASTM D792-08). Volume expansion ratio (*φ*) and void fraction (*V_f_*) were calculated using the following equations:

(1) $$\varphi =\frac{\rho_{u}}{\rho_{f}}=\frac{1}{1-V_{f}},$$

(2) $$V_{f}=\frac{\left(\rho_{u}-\rho_{f}\right)}{\rho_{u}}\times 100.$$

Image processing was carried out using ImageJ software (Version 1.51, 2018) developed by the National Institute of Health, Bethesda, MD, USA. The cell density was calculated from the SEM micrographs using the equation bellow:

(3) $$\mathrm{Cell}\;\mathrm{density}={\left(\frac{nM^{2}}{A}\right)}^{3/2}\times \varphi ,$$

where *n* is the number of cells in the micrograph and *A* and *M* are the area and magnification factor, of the micrograph, respectively.

Differential scanning calorimetry (DSC, DSC 214 Polyma, Netzsch, Germany) was performed for thermal analysis. The samples were first cooled down from ambient temperatures to −40 °C and then heated up from −40 to 240 °C. They were kept at that temperature for 5 min, before cooling down to ambient temperature again. The heating and cooling rates were 10 °C/min and all scans were under a nitrogen atmosphere, with sample sizes of ~8–9 mg. The solid TPU and TPU/PZT samples were pressed without expandable microspheres to avoid expansion during the DSC test. They were pressed at 160 °C to have the same thermal history as that of the foamed samples. Moreover, unlike foamed samples, the solid samples were not quenched in water to resemble their actual process.

The dielectric permittivity of the samples was measured using a Hewlett Packard 4192A LF impedance analyzer (Hewlett Packard, Palo Alto, CA, USA) with 16451B dielectric test fixture over the frequency range of 10 kHz to 10 MHz. The following equation was used to obtain *ε_r_*:

(4) $$\varepsilon_{r}=\frac{tC_{p}}{A\varepsilon_{0}},$$

where *ε*_0_ is the space permittivity (8.854 × 10^−12^ F/m), *C_p_* is the measured capacitance, *A* is the contact area of the electrode, and *t* is the thickness of the test sample. 

### 2.5. Permittivity Model for Ternary System

In order to better understand the dielectric response of the composite materials, experimental results were compared with the extended Yamada model to ternary systems. The Yamada model predicts the permittivity of binary systems using the following equation [34]:

(5) $$\varepsilon_{c}=\varepsilon_{m}\left\{1+\frac{n_{f}V_{f}\left(\varepsilon_{f-}\varepsilon_{m}\right)}{2\varepsilon_{m}+\left(\varepsilon_{f}-\varepsilon_{m}\right)\left(1-V_{f}\right)}\right\},$$

where *ε_c_* is the *ε_r_* of the composite, *V_f_* is the volume fraction of the filler, and *n_f_* is a parameter related to the geometry of the ceramic particles. The main advantage of the Yamada model is that the geometry of non-spherical filler can be taken into account using the *n_f_* factor [5]. In this work, the Yamada model was extended for the ternary system of TPU/PZT/air, in an attempt to predict the relative permittivity of the composite foams. In this adaptation, the relative permittivity of the piezoceramic polymer composite was calculated first using Yamada model. Then, TPU/PZT was assumed as the matrix material with air as the filler for the final ternary system. The relative permittivity of the ternary system was then calculated as follows:

(6) $$\varepsilon_{c,t}=\varepsilon_{c}\left\{1+\frac{n_{v}V_{v}\left(\varepsilon_{v-}\varepsilon_{c}\right)}{2\varepsilon_{c}+\left(\varepsilon_{v}-\varepsilon_{c}\right)\left(1-V_{v}\right)}\right\},$$

where *ε_c,t_* is the relative permittivity of the ternary system (i.e., the PZT/TPU/air composite foam), *ε_c_* is the permittivity of PZT/TPU composite obtained from Equation (5), *n_v_* is the shape factor related to the geometry of the voids, *V_v_* is the volume fraction of air phase (voids), and *ε_v_* is the relative permittivity of air (*ε_v_* = 1).

## 3. Results

### 3.1. Microstructure

**Figure 2:**
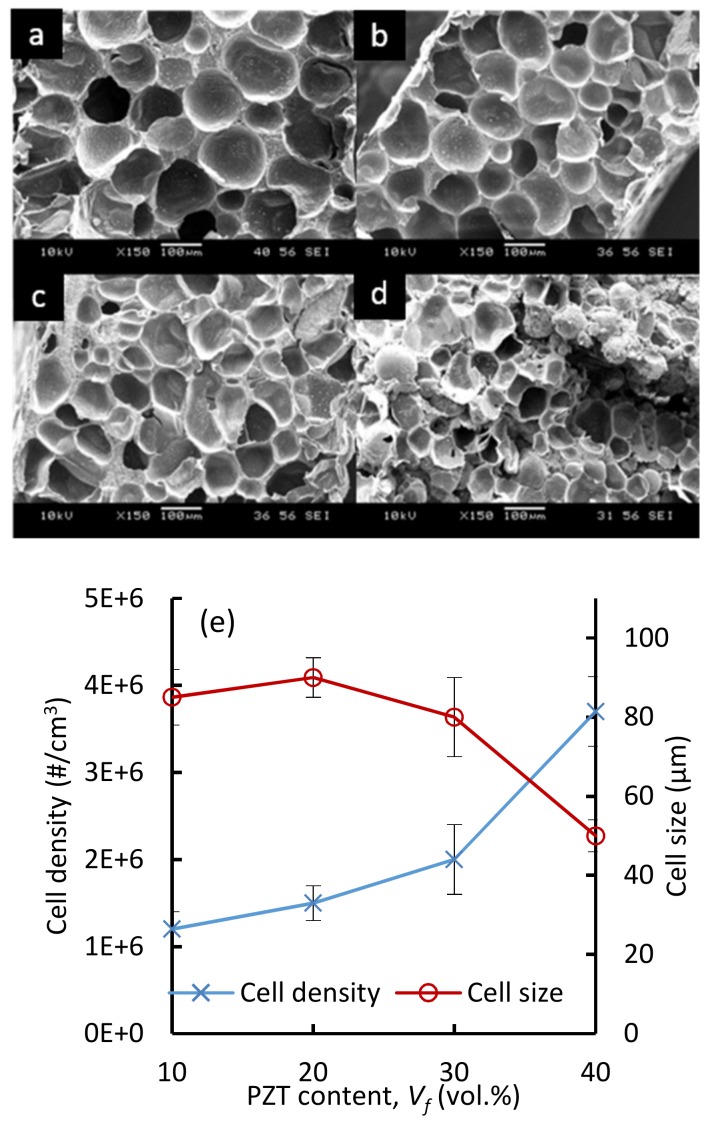
Scanning electron microscope (SEM) micrographs of TPU/PZT composite foams containing (**a**) 10 vol %, (**b**) 20 vol %, (**c**) 30 vol %, (**d**) 40 vol % PZT, and (**e**) average cell size and cell density vs. PZT content, at an expansion ratio of *ϕ* = 2.

Figure 2a–d depict the cellular morphology of composite foams at *φ* = 2, and the effect of PZT content on the cell size and cell density of the foams is summarized in Figure 2e. Foams with expansion ratios of up to *φ* = 9 were achieved using expandable microspheres. However, beyond *φ* = 4, the mechanical properties and the structural integrity of the highly-expanded composites seemed insufficient and were not further investigated. Overall, the cell size in all the foams remained between 50 and 100 μm. The average cell size changed only slightly from 85 ± 7 μm in TPU/10 vol % PZT (Figure 2a) to 90 ± 5 μm in TPU/20 vol % PZT (Figure 2b). The average cell size, however, visibly decreased to a lower value of ~50 ± 8 μm in TPU/40 vol % PZT foams (Figure 2d). This can be explained by the higher viscosity of the composites in higher filler loadings, which can restrain the cells from growing further. The cell density was slightly increased with PZT content, which compensated for the decreased cell size at a constant expansion ratio. The average cell density was calculated to be 1.2 × 10^6^ at 10 vol % PZT, increasing to 3.7 × 10^6^ at 40 vol % PZT. In this study, the microspheres content was fixed at 5 wt %, which results in an almost uniform cell density throughout all samples. In order to achieve a higher cell density (and smaller cell sizes), the expandable microspheres content can be increased. In addition to having a larger number of cells, this would result in the obstruction of the individual cells from growing, resulting in a smaller average cell size [33].

**Figure 3:**
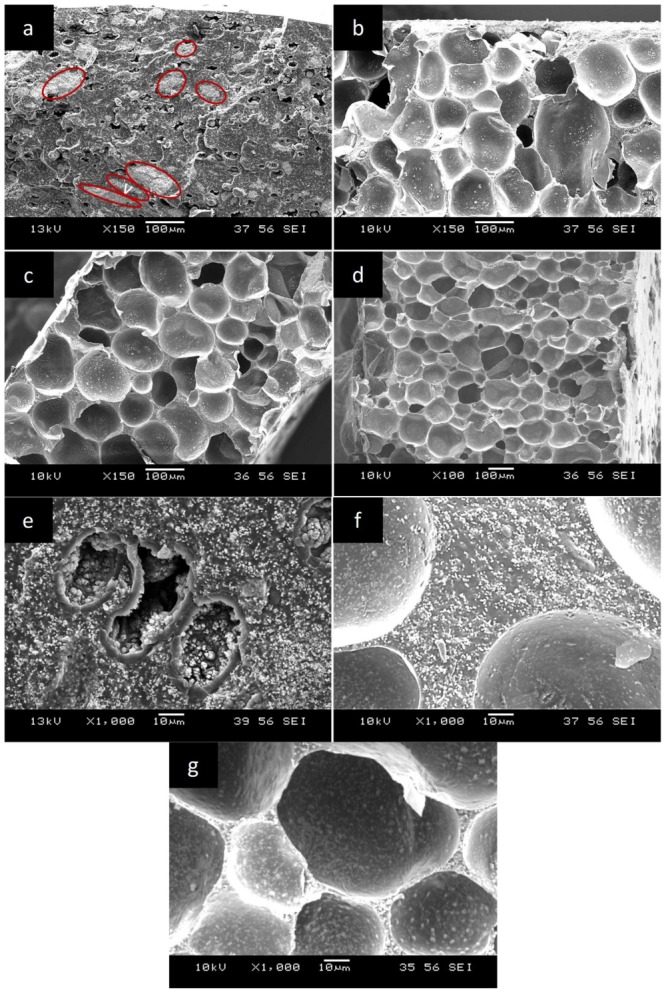
Cellular morphology of TPU/20 vol % PZT composite foams with (**a**) *φ* = 1 (solid), (**b**) *φ* = 2, (**c**) *φ* = 3, and (**d**) *φ* = 4. High magnification micrographs showing the dispersion of PZT in (**e**) solid, (**f**) cell struts, and (**g**) cell walls of foamed composites.

Figure 3a–d compare the morphology of TPU/20 vol % PZT composites in four different expansion ratios at ×150 magnification. Figure 3e–g also exhibit the PZT particle dispersion in the solid and foamed samples at ×1000 magnification. The oval-shaped whiter regions identified by red ellipses in Figure 3a are the agglomerates of PZT particles, with sizes ranging from several tens of micrometers to a few micrometers. Individually dispersed PZT particles in the solid samples are also clearly seen in Figure 3e. The agglomerates were not perfectly spherical, elongated slightly in the horizontal direction, giving them a non-unity aspect ratio (*AR*), with some in-plane orientation. As will be discussed later, the best fit to the Yamada model was obtained with an *AR* = 0.5 for solid samples. The agglomerates were distributed between the fully dispersed PZT particles, with a particle size of ~1 μm (Figure 3e). Moreover, some black voids are seen in the microstructure of solid samples (Figure 3a,e), which are the prints of unexpanded microsphere particles (~25 μm original diameter), separated from the surface during cryogenic fracture for SEM sample preparation. 

As can be seen from Figure 3c,d, at a given PZT content, with an increase in the foaming time, a greater number of expandable microspheres were activated, rather than further enlargement of the already activated microspheres. This resulted in an increased expansion ratio due to increased cell density, while the cell size remained approximately unchanged. The PZT dispersion in the foamed samples is shown in Figure 3f,g for the cell struts and cell walls, respectively. It can be seen that the PZT particles exhibited a better dispersion state inside the TPU matrix of foamed samples, and no large agglomerates were present. The better dispersion of PZT particles in the foamed samples compared with the solid ones is attributed to the effect of cell expansion. During foaming, as the microspheres enlarge, they apply local biaxial stretching and uniaxial compression on the material surrounding the cell, as shown in the literature [35,36]. This deformation could help the PZT agglomerates to redistribute and obtain a better dispersion state. As no further elongated agglomerates were present in the foamed samples, the aspect ratio of individual PZT particles, which is about one, was used in the Yamada model of foamed samples. 

### 3.2. Thermal Properties

#### 3.2.1. Crystallization

**Figure 4:**
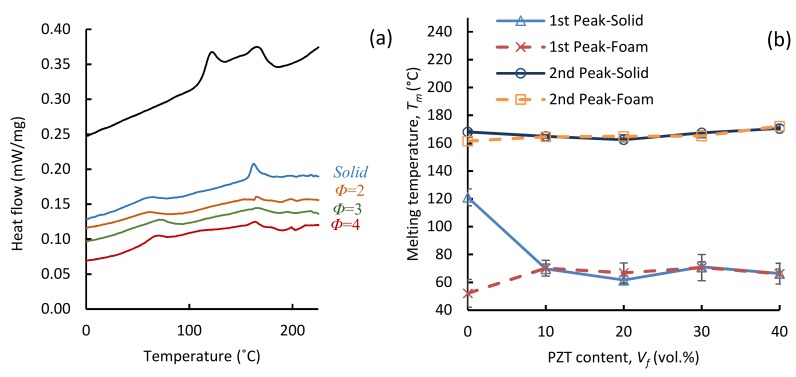
(**a**) Differential scanning calorimetry (DSC) heating thermographs of neat TPU and solid and foamed TPU/20 vol % PZT samples and (**b**) variation of the low and high melting peaks (*T*_mlow_ and *T*_mhigh_) of solid and foamed (*φ* = 2) composites as a function of PZT content.

Figure 4a depicts the DSC thermographs of neat TPU and TPU/20 vol % PZT composites with expansion ratios of 1, 2, 3, and 4. All the samples exhibited a multiple-peak melting behavior, which has been reported in earlier studies for TPU-based materials [37,38]. Neat TPU showed a relatively narrow endotherm with two distinct peaks at 121.1 and 168.1 °C; the heat of fusion for the first peak was Δ*H* = 1.54 J/g and that for the second peak was Δ*H* = 1.69 J/g. The narrow peaks are most likely attributed to the relative homogeneity of the hard-segment (HS) crystallites in the neat TPU microstructure. Pramoda et al. [38] attributed the multiple-peak phenomenon to three main reasons: (a) melting/re-crystallization/re-melting during DSC heating, (b) polymorphism, and (c) variation in morphology. During heating, the smaller and less perfect crystals started melting first, as reflected in the first melting peak, which occurred at lower temperatures (*T*_mlow_). As heating continued, the bigger and more perfect crystals then began to melt. 

Figure 4a also shows the heating thermographs of the TPU/20 vol % PZT solid and foamed composites. All the composites showed a broad endotherm with two melting peaks. A major change in the thermographs of the composites, compared with that of the neat TPU, is the shift of the first melting zone to lower temperature ranges. This is also seen in Figure 4b, where the changes in the first and second melting peaks (*T*_mlow_ and *T*_mhigh_) of the solid and foamed samples, at expansion ratio of 2, are given. Similar values of *T*_mhigh_ were obtained for both solid and foamed samples, and it appeared to be relatively independent of the PZT content and expansion ratio. This consistency in the *T*_mhigh_ values suggests that the compression molding and foaming temperature (160 °C) was not high enough to fully melt the larger and more perfect crystals. The crystallites that comprise the second melting peak (*T*_mhigh_) are larger with higher melting points. 

Neat TPU and solid samples had identical process histories. The exposure to high temperatures only occurred during the compression molding (160 °C). However, foamed samples underwent a slightly different process. The solution cast samples were first pressed at 130 °C (before foaming) and then at 160 °C for foaming. The only difference between the process histories of foams with various expansion ratios is that the foaming time was slightly higher for samples with higher expansions. It can be seen from Figure 4b that the *T*_mlow_ and *T*_mhigh_ were only affected by the introduction of PZT particles; different expansion ratios and a change in the PZT contents did not significantly affect the melting peak temperatures.

As seen in Figure 4b, in the solid samples, *T*_mlow_ decreased as the PZT was added. At the pressing temperature of 160 °C, the less perfect crystals of the hard segment were molten. Upon cooling, the presence of heavy PZT particles hindered the motion of the molten chains and prevented the full re-stacking and re-crystallization. Therefore, a lower *T*_mlow_ was obtained in the solid TPU/PZT compared with that in the solid neat TPU.

In the foamed neat TPU samples, the quenching after foaming resulted in a much faster cooling compared with the air cooling of the solid neat TPU. Therefore, the molten hard segment chains did not find enough time for re-stacking and full crystallization in the foamed TPU. This resulted in smaller and less perfect crystals and thus a significant drop in the *T*_mlow_ of the foamed neat TPU compared with that of the solid neat TPU. However, the *T*_mlow_ increased again with the introduction of PZT to foamed samples. The major reason for this increase comes from the foaming time. TPU/PZT composites had a much higher viscosity compared with neat TPU. Therefore, longer foaming times were needed to obtain a certain expansion ratio. The longer foaming time associated with the composites facilitated better re-stacking of the chains and further crystal growth. Therefore, a higher *T*_mlow_ was obtained for TPU/PZT foams compared with the TPU foam. It is believed that 10 vol % PZT was a sufficient amount of filler loading in terms of its effect on the crystal stacking, and thus a further increase of the PZT loading from 10 vol % to 40 vol % did not significantly change the *T*_mlow_. 

**Figure 5:**
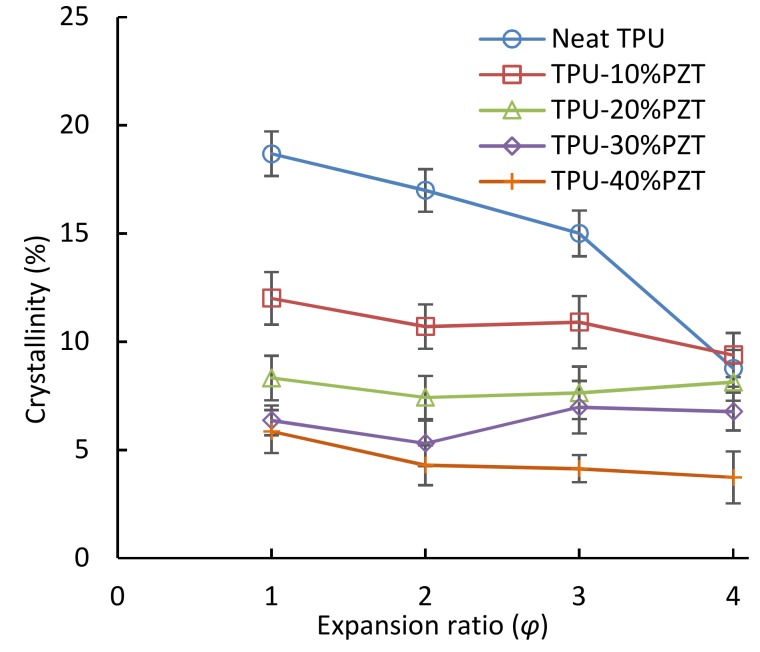
Total crystallinity of the TPU and TPU/PZT solid and foamed composites.

Figure 5 shows that the total crystallinity of the TPU’s hard segment decreases with an increase in the PZT filler content. The crystallinity is usually expected to increase with the addition of a small amount of fillers, because they could act as crystal nucleating agents and initiate more crystallization [39]. However, in this work, the TPU matrix is filled with a high content of heavy and relatively large PZT filler, and thus the viscosity is increased, causing the limited mobility of the chains. The chain mobility is further limited by an increase in the PZT content. This hinders the stacking of the TPU’s hard segment chains during pressing and cooling, and hence a decrease in the degree of crystallinity is observed with an increase in the PZT filler.

It is also noted that the total crystallinity decreased as a function of the expansion ratio in neat TPU. In order to achieve a higher expansion ratio, a longer foaming time was used. A longer time at a relatively high temperature of 160 °C for neat TPU with a lower viscosity causes a larger number of more perfect crystals (i.e., crystals associated with *T*_mhigh_) to melt. Therefore, the total crystallinity was decreased by more than 50% in the foams with the highest expansion of 4, compared with the solid ones. However, the crystallinity remained approximately unchanged with expansion ratio in the TPU/PZT composites. This is most likely the result of the dominating effect of the heavy and abundant PZT fillers, which made it difficult for the crystals to reform once they were molten.

### 3.3. Dielectric Properties

#### 3.3.1. Broadband Relative Permittivity

**Figure 6:**
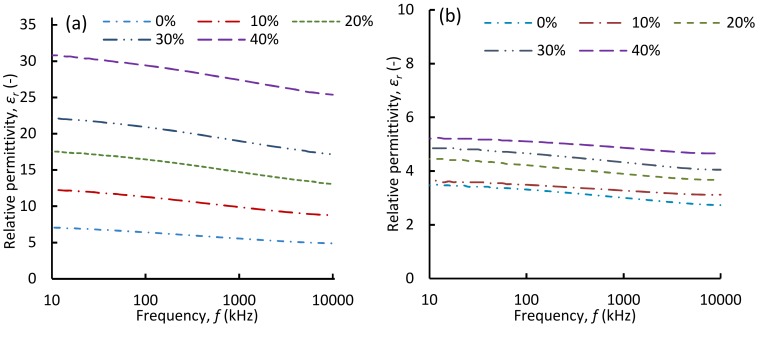
Broadband relative permittivity of (**a**) solid (**b**) foamed at *φ* = 2 of TPU/PZT composites at various PZT loadings up to 40 vol %.

Figure 6a shows the broadband *ε_r_* of TPU/PZT composites over the frequency range of 10 kHz–10 MHz at several PZT contents. The average *ε_r_* of TPU and PZT was 6.4 and 1850 (~300 times that of TPU), respectively. A homogeneous distribution of the PZT particles resulted in a continuous increase in *ε_r_* of the composites, as the high-permittivity PZT loading was increased. Overall, the permittivity values for a given PZT content were relatively frequency independent. The frequency independency decreased slightly as the PZT content increased, being most noticeable for TPU/40 vol % PZT composite. The frequency dependency of a composite permittivity can be affected by the polarization of the matrix, the filler, and the interface [40,41]. TPU and PZT have frequency independent permittivity over the tested frequency range. In addition, as neither PZT nor TPU are electrically conductive, only a few nomadic charge carriers could be accumulated at the interface of the two. Therefore, the total resultant permittivity behavior of the composites was relatively frequency independent and the slight increase of the dependency with increased PZT content is attributed to the increased interface between TPU and PZT. The measured broadband permittivity values are consistent with the data reported in the literature [42,43]. The relative permittivity values at 1 MHz were used for further analysis.

Figure 6b shows the broadband *ε_r_* of the foamed composites at *φ* = 2. The overall broadband behavior of the foamed samples was similar to that of the solid composites, with some reduced dependency on the frequency. In the foamed composites, the sensitivity of the permittivity to PZT content was lower compared with that in the solid samples. As will be discussed later, this is the result of having the third phase (gas) with a low permittivity and relatively large volume fraction.

#### 3.3.2. Impact of PZT Content and Foaming on Relative Permittivity

**Figure 7:**
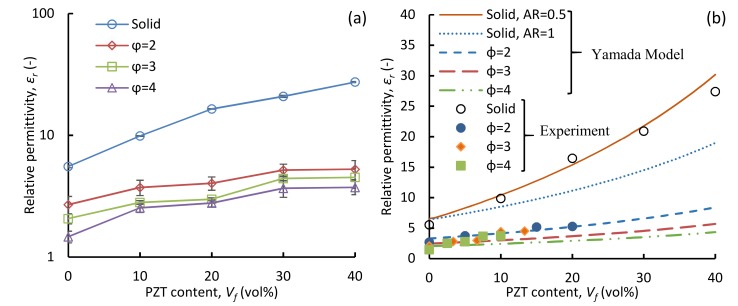
(**a**) Relative permittivity of TPU/PZT composites as a function of PZT content at the expansion ratios (void fractions) of *φ* = 2 (*V_f_* = 0.5), *φ* = 3 (*V_f_* = 0.67), *φ* = 4 (*V_f_* = 0.75). (**b**) Relative permittivity vs. PZT content for solid and foam TPU/PZT samples, compared with the prediction of the Yamada model.

Figure 7a shows the variation of *ε_r_* of TPU/PZT foams with PZT content at several fixed expansion ratios. The relative permittivity of the solid samples increased drastically with the increasing PZT content. This increase indicates a good coupling between the filler particles and polymer matrix [44]. For foamed samples, in general, the curves shifted downwards as the expansion ratio increased, demonstrating an inversely proportional relation between *ε_r_* and the void fraction. Also, the void fraction effect was pronounced as the PZT content increased. Overall, *ε_r_* decreased by approximately 50% to 85% by increasing the expansion ratio to 4. For foamed samples, *ε_r_* increased as PZT content increased, but it was not as significant as solids, which can be attributed to the dominance of low-permittivity gas phase at high void fractions. In addition, a further increase of the expansion ratio decreased *ε_r_*. 

Figure 7b compares the results of the experiments for both solids and foams with the Yamada model. The Yamada model results for the solid samples, using two different aspect ratios of PZT particles, are also shown in Figure 7b. The volumetric contents for the foamed composites were modified in order to obtain final volume fractions of the constituents needed for the model. For this, the volume occupied by the voids was subtracted from the overall volume, and the new PZT contents were calculated based on the available TPU/PZT material in the foams. The relative permittivity of TPU (*ε_1_*) and PZT (*ε_2_*) used in the model was 6.4 and 1850, respectively. Assuming perfectly spherical particles (*AR* = 1), the Yamada model underestimates *ε_r_* values and the discrepancy increases with the increase in the piezoelectric particle volume fraction. Babu et al. [44] related this increasing discrepancy to the diversion from the original spherical shape of the particle and leaning towards an ellipsoidal shape. As discussed, Figure 3g schematically represents the dispersion of PZT particles in the solid samples and can be described as a matrix with particles having a non-unity AR. When using the Yamada model, the shape-dependent parameter *n_f_* in the Equation (5) needs to be modified to achieve the best fit to the experimental values [34,45]. In this work, an *AR* ≃ 0.5 was found to best fit the experimental results of solid composites. *n_f_* was thus selected such that that it represents the *AR* ≃ 0.5 for the solid samples. With this assumption, the predicted values from the Yamada model match well with the experimental data for the entire PZT content range tested. 

Figure 7b also shows the comparison of the experimental data with the Yamada model for the foamed composite samples. The extended Yamada model was used to predict the permittivity of TPU/PZT/gas composites. For the ternary system, the aspect ratio for both PZT and air particles was assumed to be 1, which is a reasonable assumption, according to the SEM micrographs of the composites, as the PZT particles are mostly individually dispersed and the voids are relatively spherical. A relatively good agreement was obtained between the experimental values and the Yamada model predictions for all the TPU/PZT foams, indicating the capability of the Yamada model to predict *ε_r_* of ternary systems. 

**Figure 8:**
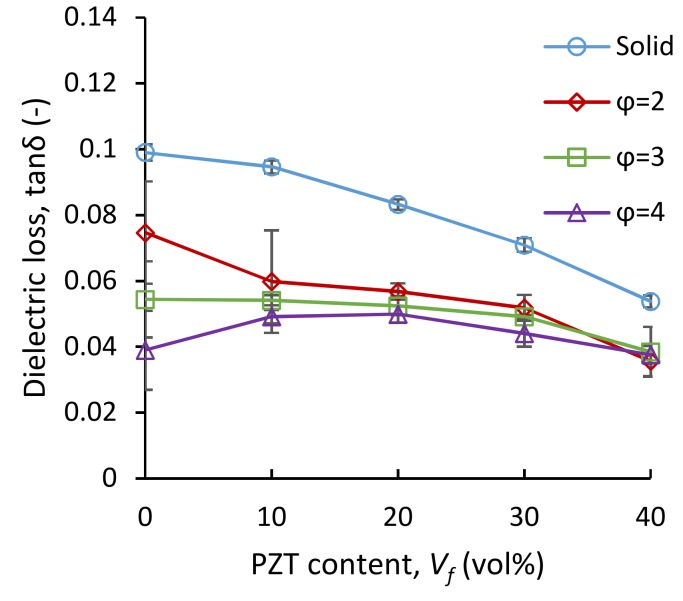
Dielectric loss of TPU/PZT composites as a function of PZT content at the expansion ratios of *φ* = 2 (*V_f_* = 0.5), *φ* = 3 (*V_f_* = 0.67), and *φ* = 4 (*V_f_* = 0.75).

Figure 8 gives the dielectric loss (tan*δ*) of the solid and foamed samples as a function of PZT loading. The dielectric loss of the used TPU and PZT was 0.1 and 0.016, respectively. In the solid samples, the dielectric loss continuously decreased from 0.1 in neat TPU to 0.055 in TPU 40 vol.% PZT composites, as the tan *δ* of PZT is ~6 times smaller than that of TPU. In the foamed samples, at an expansion of two (*φ* = 2), the dielectric loss curves for all PZT loadings shifted downwards, and an overall decreasing trend of the dielectric loss versus PZT content was present throughout all the samples at *φ* = 2. Neat TPU showed the biggest decrease after foaming, as tan*δ* dropped from 0.1 for solid to ~0.04 with 75% foaming (*φ* = 4). In the composites, overall, the value of tan*δ* decreased only slightly by further expansion beyond *φ* = 2. The rate of tan*δ* drop as a function of expansion ratio reduced as the PZT content was increased in the composites. This was the result of the reduced content of TPU, which had the highest dielectric loss. 

## 4. Conclusions

Composite foams of TPU/PZT with different PZT contents (ranging from 10 to 40 vol.%) and various expansion ratios (*φ* =1 to 4) were fabricated. Expandable microspheres were utilized to create the cellular structure in these highly loaded composites. The morphology and cellular structure were studied. The foams demonstrated microcellular structures with relatively spherical cells at all PZT contents and expansion ratios. The DSC was performed on the samples in order to assess the effect of filler and expansion ratio on the thermal properties of the composites. All the samples showed double melting peaks, which became shorter, as the expansion ratio increased. *T*_mhigh_ values did not change after the addition of PZT filler or foaming, but *T*_mlow_ decreased by ~60 °C after the addition of PZT particles.

The dielectric permittivity, *ε_r_*, increased with an increase in the PZT content. Overall, the introduction of cellular structure decreased *ε_r_*. The permittivity exhibited an inversely proportional relation with the void fraction, and a maximum of 7.7 times decrease was obtained at an expansion ratio of *φ* = 4. At the higher expansion ratios, the relative permittivity appeared to be less dependent on PZT loading, because of the dominance of the low-permittivity gas phase. Moreover, the experimental *ε_r_* measurements showed good agreement with the predictions made by the Yamada model, extended to the ternary system of piezoceramic/polymer composite foams. An *AR* = 0.5 best described the Yamada model of the solid samples, while *AR* = 1.0 worked well for both the PZT and gas phases in the ternary system of foam. This was because of the fact that foaming helped in better dispersing the agglomerates of PZT formed during solution casting. These foams will exhibit higher voltage sensitivity compared with their solid counterparts, broadening the applications of piezocomposites.
